# Effects of a sex-ratio distorting endosymbiont on mtDNA variation in a global insect pest

**DOI:** 10.1186/1471-2148-9-49

**Published:** 2009-03-03

**Authors:** Ana M Delgado, James M Cook

**Affiliations:** 1 Division of Biology, Imperial College London, Silwood Park Campus, Ascot, UK; 2 School of Biological Sciences, University of Reading, Whiteknights, Reading, UK

## Abstract

**Background:**

Patterns of mtDNA variation within a species reflect long-term population structure, but may also be influenced by maternally inherited endosymbionts, such as *Wolbachia*. These bacteria often alter host reproductive biology and can drive particular mtDNA haplotypes through populations. We investigated the impacts of *Wolbachia *infection and geography on mtDNA variation in the diamondback moth, a major global pest whose geographic distribution reflects both natural processes and transport via human agricultural activities.

**Results:**

The mtDNA phylogeny of 95 individuals sampled from 10 countries on four continents revealed two major clades. One contained only *Wolbachia*-infected individuals from Malaysia and Kenya, while the other contained only uninfected individuals, from all countries including Malaysia and Kenya. Within the uninfected group was a further clade containing all individuals from Australasia and displaying very limited sequence variation. In contrast, a biparental nuclear gene phylogeny did not have infected and uninfected clades, supporting the notion that maternally-inherited *Wolbachia *are responsible for the mtDNA pattern. Only about 5% (15/306) of our global sample of individuals was infected with the *plutWB1 *isolate and even within infected local populations, many insects were uninfected. Comparisons of infected and uninfected isofemale lines revealed that *plutWB1 *is associated with sex ratio distortion. Uninfected lines have a 1:1 sex ratio, while infected ones show a 2:1 female bias.

**Conclusion:**

The main correlate of mtDNA variation in *P. xylostella *is presence or absence of the *plutWB1 *infection. This is associated with substantial sex ratio distortion and the underlying mechanisms deserve further study. In contrast, geographic origin is a poor predictor of moth mtDNA sequences, reflecting human activity in moving the insects around the globe. The exception is a clade of Australasian individuals, which may reflect a bottleneck during their recent introduction to this region.

## Background

Patterns of within-species variation in animal mtDNA are influenced by various factors, including mutation, selection, demography and geography, and analysis of haplotype diversity patterns can provide information on population structure and gene flow. In addition, mtDNA sequences are often used to investigate the evolutionary history of a species, by combining geographic and phylogenetic information in phylogeographic studies [1]. However, theory predicts that mtDNA variation and evolution may also be influenced substantially by endosymbionts that are maternally co-inherited with the mitochondria [2]. In insects, maternally-inherited intracellular *Wolbachia *bacteria are of particular concern, because they have been detected in hundreds of species [3] and are estimated to infect about 2/3 of all insect species [4]. *Wolbachia *cause various modifications of host reproductive biology, including cytoplasmic incompatibility (CI), parthenogenesis, feminisation and male-killing [5,6]. In addition, a single infection can be responsible for causing more than one phenotype, e.g. CI and male-killing [7]. *Wolbachia *enhance their own spread through the host population by means of these phenotypes and, in so doing, drive the associated host mitochondrial haplotypes to high frequencies, causing dramatic changes in host mtDNA patterns [8]. Consequently, analysis of variation in insect mtDNA should take account of possible endosymbiont effects whilst attempting to uncover and explain patterns in terms of host ecology [2,8].

Here, we explore the role of *Wolbachia *bacteria in explaining patterns of mtDNA variation in the diamondback moth (*Plutella xylostella*), a global pest of *Brassica *plants (cabbages and relatives). A previous study [9] reported the occurrence of two different *Wolbachia *isolates in two different *P. xylostella *individuals during a general screening of diverse insects for *Wolbachia *infections; however, there have been no wider studies of the occurrence of these isolates in *P. xylostella *or of their effects on host phenotypes and genetic variation. Consequently, we screened individuals from four continents for *Wolbachia *infections and then investigated the correlation between infection with the dominant isolate (*plutWB1) *and mtDNA variation. We also sequenced a biparentally inherited nuclear gene marker, whose phylogeny should reflect demography and geography, but not any *Wolbachia*-associated driving of cytoplasmic factors. In addition, we conducted experiments to test whether *plutWB1 *produces a phenotypic effect on the host that is likely to drive the infection, and its associated mtDNA haplotype, through local populations.

The diamondback moth is amongst the most globally distributed of all Lepidoptera [10]. It is generally thought to be endemic to the Mediterranean region [11], although an African origin has also been suggested [12]. Its current global distribution is due to two processes – natural migrations and man-made introductions. Direct observations using vertical-looking radar have confirmed that long distance migration from continental Europe is the source of populations in the UK in late spring [13]. Meanwhile, transport to many disparate locations across the globe is likely to be the result of accidental introductions during vegetable shipments [14]. Consequently, we predict an unusually weak correlation between geographic and genetic (mtDNA) divergence in *P. xylostella *because recent human activity has moved moths between continents.

In summary, we screened *P. xylostella *samples from four continents in order to: (1) determine the global diversity and prevalence of *Wolbachia *infections, (2) test whether *Wolbachia *infections were associated with particular mtDNA haplotypes, (3) test whether *plutWB1 *produces a drive phenotype, 4) test for a breakdown of the usual strong link between geographic continent of origin and mtDNA sequence variation, and 5) compare mtDNA results with those from a nuclear marker that should not have its phylogeny shaped by cytoplasmic drive mechanisms.

## Results

### Global screening for *Wolbachia *in *P. xylostella*

**Figure 1:**
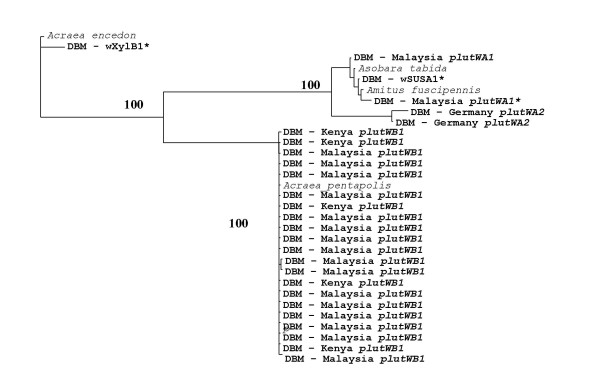
**Phylogeny of *Wolbachia *sequences from *P. xylostella *hosts**. Neighbour-joining tree of *Wolbachia wsp *sequences from *Plutella xylostella *(country of origin) and other insects. Bootstrap values >65% are shown. * denotes *Wolbachia *isolates found infecting *P. xylostella *in a previous study [9]. Sequences have been deposited in Genbank with accession numbers EU833334–833358.

In order to explore the prevalence and diversity of *Wolbachia*, we screened 306 individuals from 10 countries on four continents (Table 1) and isolated three *Wolbachia *isolates, termed *plutWA1*, *plutWA2 *and *plutWB1 *(Figure 1). The first two isolates were extremely rare and found in only two individuals each, while *plutWB1 *was found in about 5% (15/306) of the global sample of individuals (Table 1). We detected *Wolbachia *only in individuals from Malaysia, Kenya and Germany. However, even in these countries, most individuals were uninfected (Table 1).

**Table 1:** Global screening of *Wolbachia *infections in *Plutella xylostella*.

**Country**	**N screened**	**N infect. (%)**	**Infections**
Malaysia	122	10 (8.2)	plutWB1, plutWA1
Australia	73	0	
Kenya	41	5 (12.2)	plutWB1
UK	24	0	
S Africa	6	0	
Taiwan	10	0	
Hawaii	10	0	
Sweden	5	0	
Germany	5	2 (40)	plutWA2
New Zealand	10	0	

Total	306	15	

### Moth mtDNA phylogeny

**Figure 2:**
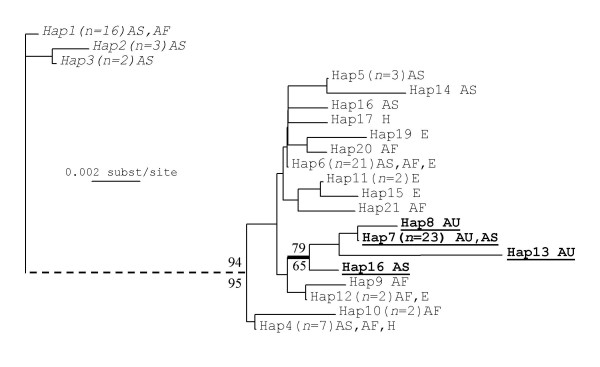
**Phylogeny of *P. xylostella *moths using mtDNA data**. Neighbour-joining tree of *P. xylostella *mtDNA (CO1) haplotypes. Numbers in brackets show individuals with a given haplotype. Geographic occurrences are shown as Asia (AS), Africa (AF), Australasia (AU), Europe (E) and Hawaii (H). The broken line shows that the long branch between Clade A (italics; all 21 individuals infected with PlutWB1) and Clade B (normal text; all 71 not infected) is not to scale. A further Clade C (all 26 individuals from Australasia) is shown in bold. Sequences have been deposited in Genbank with accession numbers EU833237–833257.

We obtained mtDNA sequences for 92 *P. xylostella *individuals and three members of the closely related outgroup species *Plutella porrectalla *(Table 1). The mtDNA fragment analysed consisted of 637 bp of the CO1 gene. There were 72 variable nucleotide sites, of which 55 were informative for maximum parsimony (MP) analyses. This variation yielded 22 discrete haplotypes (Figure 2), of which 11 where unique to one individual.

MP and neighbour-joining (NJ) phylogenies were very similar and revealed a deep split (2.5% sequence divergence) between all individuals infected with *plutWB1 *and all those that were not (Figure 2). In contrast, the largest pairwise distance between any two uninfected individuals was only 1.75%. We sequenced 21 individuals from Kenya and Malaysia that were infected with *plutWB1*. They all had very similar COI sequences, with maximum pairwise divergence of only 0.314%, forming haplotypes 1–3 and a well-supported monophyletic clade (Figure 2). This supports a single origin of the infection and we further tested for an association between mtDNA haplotypes and *plutWB1 *by randomising the infection status of individuals across the tips of the phylogeny and calculating the minimum number of changes in infection status implied (see [15], for a similar application). All of the 100 randomisations implied more than the one change observed in the actual data set, providing significant support (P < 0.01) for the association between infection status and haplotype that is apparent by eye. In contrast to the clear pattern found with this isolate, the two A clade *Wolbachia *isolates were each found in only two individuals, precluding further useful analysis.

In general, individuals do not cluster on the phylogeny according to their geographic origin. However, all individuals from Australia (Haplotypes 7, 8 and 13) and New Zealand (Haplotype 7), along with one from Malaysia (Haplotype 7) form a well-supported monophyletic clade (Figure 2). Australasia was the best sampled region, with individuals from 14 sites spanning most of Australia, as well as two sites in New Zealand.

### MtDNA haplotype analysis

Haplotype and nucleotide diversity estimates for different geographic regions are presented in Table 2. Nucleotide diversity (π) for each region is generally low but ranges from 0.0013 to 0.091. The highest nucleotide diversity was found in Asia, followed by Africa; importantly, these are the two regions that harboured the *plutWB1 *infection and its associated divergent haplotypes. Australasia showed the lowest haplotype diversity and nucleotide diversity, despite field sampling from 14 sites across Australia and two in New Zealand. This is reflected further by comparisons with data from Hawaii (Table 2). Hawaii, like Australasia, is isolated by sea from other continents and was probably only colonised relatively recently by *P. xylostella *due to human travel. Only four individuals from Hawaii were sampled, but these had higher haplotype diversity than the Australasian individuals and values similar to those in Europe. All infected groups have lower haplotype number, and haplotype and nucleotide diversity, than their respective uninfected groups (Table 2).

**Table 2:** Haplotype and nucleotide diversity of CO1 sequences from different geographical regions and infection categories.

	**N**	**NHap**	**DHap**	**S**	π	**SD(π)**
**Region (sites)**						
Australasia (16)	22	5	0.338	8	0.0013	0.0007
Asia (6)	31	10	0.839	17	0.0093	0.0006
Africa (6)	14	9	0.934	15	0.0091	0.0010
Europe (5)	17	5	0.507	5	0.0012	0.0004
Hawaii (1)	4	2	0.500	2	0.0015	0.0008
						
**Group**						
All Infected	21	3	0.4	2	0.0009	0.0003
All Uninfected	71	18	0.8	22	0.00324	0.00031
Malaysia -Infected	18	3	0.508	2	0.0015	0.00032
Malaysia Uninfected	12	7	0.864	9	0.00317	0.00071
Kenya Infected	5	2	0.6	1	0.00093	0.00028
Kenya Uninfected	8	6	0.893	6	0.00237	0.00059

Key: N = No. individuals; NHap = No. Haplotypes; DHap = Haplotype diversity; S = No. of variable sites; π = nucleotide diversity.

### Moth nuclear DNA

**Figure 3:**
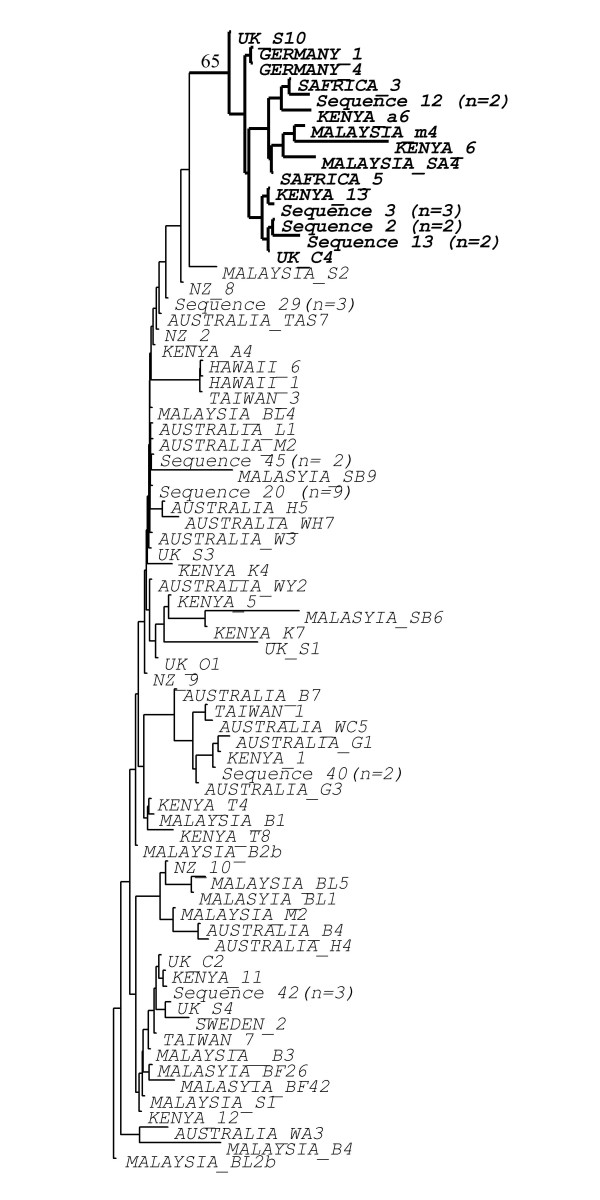
**Phylogeny of DBM moths using nuclear data**. Neighbour-joining tree of the 74 different *Plutellaxylostella *nuclear DNA (L27a gene) sequences. Numbers in brackets show multiple individuals with the same DNA sequence. The clade with bold labels and thick lines is supported by a major indel (see text) and has bootstrap support of 65. Country of origin is given for sequences found only in one moth. Sequences have been deposited in Genbank with accession numbers EU833259–833333.

We also analysed approximately 200 bp of the L27a nuclear ribosomal protein gene for the same 92 *P. xylostella *individuals. We obtained 74 different sequences (Genbank accession numbers EU833259–833333), 65 of which were unique to one individual. However, there was low sequence divergence and the vast majority of pairs of individuals differed by only 1–4 nucleotide substitutions. Further, many substitutions were unique and therefore not phylogenetically informative. Nevertheless, two clades can be defined by the presence/absence of a 9 bp indel event. Clade A contained 74 individuals from all five continents, while clade B contained 20 individuals from Europe, Asia and Africa (Figure 3). In contrast to the case for mtDNA, there were not separate infected and uninfected clades. A mixture of infected and uninfected individuals occurred in each clade. All individuals from Australasia were in clade A, indicating that Australasian populations have reduced nuclear diversity compared to most other countries (present in both clades), as suggested by Endersby [14], based on microsatellite markers.

### Sequence variation in the *Wolbachia wsp *gene

The *plutWB1 *isolate found in our study shows high sequence divergence (17.3–17.5%) from the B-clade *Wolbachia *isolate (*wXyl-B1*) previously identified infecting *P. xylostella *[9] (Figure 1). However, it has the same *wsp *sequence (0–0.2% sequence divergence) as an isolate found infecting the African butterfly, *Acraea pentapolis *[16].

### Phenotypic effects of *Wolbachia*

We found a female biased sex ratio of approximately 0.62 (proportion females) in field *P. xylostella *populations in the Cameron Highlands, Malaysia (Table 3). The sex ratio was only significantly different from 1:1 for the site with the largest sample (or using pooled data from all sites). However, the sex ratio was actually remarkably consistent (0.60–0.63) between the four sites surveyed in this region (Table 3).

**Table 3:** Sex ratios and *plutWB1 *infection frequencies in four Malaysian valleys.

**Valley**	**N**	**Prop. Female**	***χ*^2^**	**Infected (95% CI)**
Bertum	145	0.63	4.79*	0.05 (0.07)
Blue	60	0.62	1.22	0.02 (0.1)
Sungai Palas	40	0.63	0.81	0 (0.01)
Tringkapp	48	0.60	0.67	0 (0.1)

We successfully established two infected and three uninfected isofemale lines from field-caught Malaysian *P. xylostella *individuals and maintained them in the laboratory for nine generations (see methods). The two infected lines had female biased mean sex ratios (0.68, 0.66, Table 4) that were similar but slightly more biased than those observed in the field populations (see above) and these did not change significantly over nine generations of laboratory culture (*F *= 3.634, d.f. = 2, *P *> 0.05). In contrast, the uninfected lines had unbiased sex ratios (0.5, 0.54, 0.52) (Table 4). The *plutWB1 *infection appears to be a sex ratio distorter that increases the proportion of female hosts, as infected lines have significantly higher proportions of females than uninfected lines (*t *= 8.82, d.f. = 3, *P *< 0.01).

**Table 4:** Sex ratios of infected laboratory lines over nine generations.

	**Line 17**			**Line 95**		
**Gen**	**Females**	**Males**	**Prop. Fem**.	**Females**	**Males**	**Prop. Fem**.
1 to 3	73	23	0.76	253	88	0.74
4 to 6	545	218	0.71	408	242	0.63
7 to 9	209	142	0.60	224	121	0.65

The proportion of infected individuals in infected lines decreased over the nine generations (*F *= 63.66, d.f. = 2, *P *< 0.05), suggesting that vertical transmission of *Wolbachia *is not perfect. This was also found when both sexes were tested separately (Males: *F *= 26.15, d.f. = 2, *P *< 0.05 and Females: *F *= 496.19, d.f. = 2, *P *< 0.05). There was no significant difference between the sexes, with a mean of 68% of males and 74% of females infected (*t *= 1.4, d.f. = 17, *P *> 0.05).

## Discussion

### *Wolbachia *infections in *P. xylostella*

We detected three different *Wolbachia *isolates in *Plutella xylostella*. The two A-clade isolates were very rare, each occurring in only two individual moths. The B-clade isolate *plutWB1 *was more common, but, where present in Malaysia and Kenya occurred at low frequencies of about 10%. It is therefore likely that further screening in some of the other countries will also reveal *plutWB1 *(see Jiggins *et al*. 2001 for a discussion of sampling effort and *Wolbachia *detection). Interestingly, *plutWB1 *differs considerably in *wsp *sequence from a previously reported B-clade infection (*wXylB1*) of *P. xylostella *in the USA [9]. It thus appears that *P. xylostella *has acquired different *Wolbachia *infections in different parts of its now global distribution. None of these infections appears common or widespread and some may be transient.

Interestingly, the whitefly *Bemisia tabaci*, another major crop pest with an essentially global distribution, has also been found to harbour a number of different *Wolbachia *infections [17]. The *plutWB1 *infection in *P. xylostella *is present in at least two continents and these insects probably stem from a single ancestral infection event, since their mtDNA haplotypes are very similar and form a well-supported and differentiated monophyletic group within the *P. xylostella *COI phylogeny (Figure 2). There is a small amount of variation in both the COI sequences of infected individuals (Figure 2) and the *wsp *sequences of their infections (Figure 1), reflecting a small amount of evolution since the original infection event.

### Phylogenies, infection and geography

The host COI phylogeny has two major clades that correlate perfectly with presence or absence of the *plutWB1 *infection. The association between the very similar haplotypes 1–3 and *plutWB1 *infection supports the idea that *Wolbachia *have driven this haplotype group to a significant if low frequency in infected populations. Our study further suggests that this infection is a sex ratio distorter and this phenotypic effect upon the host is likely to have driven the infection and associated haplotype group into the wider population. In contrast, there is little correlation between mtDNA haplotype and geographic origin. Infected and uninfected individuals from the same site in Malaysia (or Kenya) fall into the two different clades rather than group together.

In contrast, there is little tendency for individuals from the same country or even continent to cluster together in the mtDNA phylogeny (Figure 2). However, the lack of this pattern is consistent with recent and repeated movements of insects between continents due to trade in cruciferous crops. It is interesting that Australasia stands out as an exception to the general pattern. We sampled many Australian populations and two from New Zealand and all individuals belong to a single clade with very little mtDNA variation. This suggests that Australian and NZ populations probably stem from a single colonisation event from Asia around 120 years ago [18] that was then transferred from Australia to NZ or vice versa. The very low mtDNA diversity could reflect a small initial founder population from SE Asia. Low variation in the L27a gene (this study) and low microsatellite diversity [14] in Australian individuals further support this idea and suggest success of stringent quarantine procedures in modern times [14].

Finally, we note that selection for resistance to pesticides and biocontrol agents [19,20] may also influence *P. xylostella *genetic variation. However, this would most likely influence both mtDNA and nuclear DNA, while our main result is a clear association of a sex-ratio distorting infection with patterns of mtDNA variation.

### Sex ratio distortion

Our data show that the *plutWB1 *infection is associated with substantial sex ratio distortion, so that females outnumber males about two to one in infected lines. The fact that infection frequency was the same in males and females in our experiments argues against it being a male-killer. However, such a low infection frequency would be surprising for a feminizing bacterium, but is not unusual for a male-killer and host resistance to male-killing can evolve, as in the butterfly *Hypolimnas bolina *[21]. Male-killing alone cannot explain the sex ratio bias in our infected lab lines and feminisation of genetic males is an interesting possibility for future study. It is also possible that a more complicated scenario could apply and that *Wolbachia *is not, or not the only, agent of sex ratio bias [22]. Our evidence for the role of *Wolbachia *is currently only correlational, which is an important but not definitive line of evidence [22]. Recent work has revealed that other less common, but still widespread [23] insect endosymbionts can cause similar hosts effects to *Wolbachia *[23,24]. Furthermore, detailed analysis of a case involving *Ostrinia *moths, initially thought to involve a feminising *Wolbachia *isolate, has revealed complex fitness interactions between sex chromosomes and infection status leading to differential mortality by sex [25].

## Conclusion

Global patterns of mtDNA variation in the diamondback moth are shaped largely by the presence or absence of the *plutWB1 Wolbachia *infection. This occurs patchily at low frequencies and apparently distorts host sex ratios to a 2:1 bias in favour of females. The cause and mode of sex ratio distortion deserves further study. In contrast to infection status, the geographic origin of a given individual is a poor predictor of its mtDNA haplotype, due to recent human transport of insects between continents in vegetable crops.

## Methods

### Field sampling of insects on four continents

Between 2001–2006 we sampled *Plutella xylostella *individuals from 33 sites in 10 countries: Australia, New Zealand, Malaysia (Peninsular and Sabah), Kenya, South Africa, United Kingdom, Taiwan, Sweden, Germany and Hawaii. We also included three *Plutella porrectella *individuals from the UK as outgroups. Insects used were either final instar larvae or adults and were field caught individuals or their F1 offspring (except Sweden, South Africa and UK – Oxford, which were laboratory cultures). Insects were stored in 70–100% ethanol for preservation of DNA.

### DNA extraction

To minimise the risk of contamination, the pre-extraction treatment and all DNA extractions were performed under sterile conditions After taking insects out of their collection tubes, each individual was cleaned by immersion in 70% ethanol, followed by two rinses in double-distilled DNA/RNA free water and then allowed to dry for 5 min. Larvae were dissected and checked for endoparasites. DNA was then extracted by grinding the abdomen of adults, or the entire larva, in 200 ul of 5% Biorad Chelex 100 resin solution in the presence of proteinease K (12μg/μl), followed by 3 h incubation at 55°C and 15 min boiling at 96°C. The samples were then centrifuged and stored at -20°C until use. For each set of extractions a blank extraction was performed using all the reagents minus the DNA extract (this was performed at least once for every 10 insects).

### *Wolbachia *screening and sequencing

We tested 306 individuals for the presence of *Wolbachia *infection with PCR using extended versions of the *Wolbachia *specific primers *ftsZf1 *5' GTT GTC GCA WTA CYG ATG CTC A 3' and *ftsZr1 *5' CTT AAG TAA GCT GGT ATA TCA ATA 3' [26] to amplify an approximately 1000 bp stretch of the *FtsZ *gene encoding the bacterial cell cycling gene. We scored individuals yielding a product of the expected size as provisionally infected, and samples that did not amplify as provisionally uninfected.

DNA extracts that tested positive for *ftsZ *were then double-checked by performing a PCR using a different set of *Wolbachia *specific primers (*wsp81F *and *wsp691R*) [27], which amplify the *wsp *(*Wolbachia *Surface Protein) gene and yield a product of approximately 550 bp. All samples that were positive for *ftsZ *also tested positive using the *wsp *primers. We later sequenced these *wsp *products.

Provisional negative samples were tested for the quality of DNA extract by performing PCR for part of the insect mitochondrial cytochrome oxidase I (CO1) gene. We used C1-J-2183 (alias Jerry) and L2-N-3014 (alias Pat) primers, which yield an approximately 1000 bp product [28]. If CO1 PCR was successful, we regarded the sample as uninfected. If it failed, we excluded the sample from our data. DNA extracts that scored as *Wolbachia *infected were also amplified for the CO1 gene. All the CO1 genes amplified were later sequenced.

Finally, a segment of the nuclear L27a gene, encoding the ribosomal protein L27a was amplified using the primers *L27aFor1 *5' ACG GTC ATG GAC GTA TCG GTA A 3' and *L27aRev2 *5' ATG TTG ATG ACT GGC ACC TTG C 3' (S. Baxter, *pers. comm*.) to yield an approximately 200 bp product, which was later sequenced.

The PCR temperature profile for *ftsZ *was 95°C for 30 sec, 55°C for 1 min and 72°C for 1 min, for a total of 35 cycles, and final elongation time of 7 min at 72°C. We used the same conditions for *wsp*, L27a and CO1 (annealing temperature reduced to 50°C). We electrophoresed 25 ul of each PCR product on a 1% agarose gel to determine amplicon presence and size. We excised gel bands and purified them using a GFX DNA Purification Kit (Amersham Pharmacia Biotech Inc.), before sequencing directly in both directions using the PCR primers. Sequences were obtained using the ABI PRISM Big Dye Terminator Cycle Sequencing Kit (Perkin Elmer Inc.) and the ABI Prism 3700 DNA Analyser (Perkin Elmer Inc.) and assembled using Sequencher™ (Gene Codes Cooperation).

### Phylogenetic analysis

Sequences were aligned manually using SE-AL v2.0a11 Carbon [following previous alignments and excluding the third hypervariable region in the case of *wsp *[9,27,29]]. There were no gaps in the CO1 sequence alignment of *P. xylostella *samples. There was a 9 bp gap that started at base 182 in the L27a sequence alignment. All phylogenies were estimated using PAUP* version 4.0b [30].

To determine the phylogenetic affinities of the *Wolbachia *isolates we constructed a neighbour-joining tree [31] of the *wsp *sequences. We adopted the standard criterion of 2.5% *wsp *sequence divergence to distinguish between *Wolbachia *isolates [27]. Isolates differing by this amount should be discrete infections, although recent work shows that infections with similar sequences in one gene may differ considerably in other genes, making a multi-locus strain typing (MLST) approach necessary to show close identity of apparently similar isolates from different hosts [32,33].

MtDNA (CO1) phylogeny was reconstructed using NJ and MP methods. MP trees were reconstructed using the method of Quicke et al. [34] by conducting an initial heuristic search of 10,000 random additions, tree-bisection-reconnection (TBR) branch swapping, and holding one tree per replicate. We then used the trees generated by the initial search as starting trees for a second heuristic search, in which we saved multiple trees. We assessed clade support using 1000 bootstrap replications.

We used MODELTEST 3.06 [35] to select nucleotide substitution models for NJ trees. The model HKY+G was selected for *wsp *and CO1. Bootstrap values were generated from 10,000 replicates. We rooted the mtDNA phylogeny using the outgroup (*Plutella porrectalla*).

### MtDNA haplotype analysis

Haplotype number, haplotype diversity and nucleotide diversity (π) [36-38] were calculated for five geographic regions: 1. Australasia (Australia/New Zealand), 2. Africa (Kenya/South Africa), 3. Europe (UK, Germany, Sweden), 4. Asia (Malaysia, Taiwan) and 5. Hawaii, and also for matched infected and uninfected groups (Tables 2 &3). Haplotype diversity describes the number and frequency of different haplotypes and nucleotide diversity is defined as the average number of pair-wise nucleotide difference per site. Haplotype and nucleotide diversity measurements are appropriate for this type of data because they do not depend on the length of DNA fragment or sample size [36], unlike the number of pairwise nucleotide differences or haplotypes.

### Phenotypic effects of *plutWB1*

We collected individuals from four valleys in the Cameron Highlands, Malaysia in May 2004 to obtain data on field sex ratios and insects to establish isofemale laboratory lines. We scored 293 individuals collected as last instar larvae or pupae and then reared to adulthood. The valleys are separated by forested mountain ridges and consist of farms that grow commercial *Brassica *plants and other vegetables.

We established two *plutWB1 *infected lines (17 and 95) and three uninfected ones (26, 61 and 86) from the field-sampled insects by mating a virgin male with a virgin female individual. Each line was kept in a rectangular, transparent container (116 × 8 × 7 cm L × W × D) and containing a cotton-wool ball soaked in 10% honey solution and an egg-laying sheet (tin foil, 13 × 5 cm) dipped in cabbage solution (65 g cabbage per 500 ml ddH_2_0) for 3 days. We then reared the eggs produced by each pair to the pupal stage on *Brassica napus *seedling trays (50 × 25 × 6 cm) in cages with a metal cube frame, covered in fine netting. To avoid overcrowding, no more than 100 larvae were reared per seedling tray.

To continue each isofemale line, pupae were collected and placed in individual tubes and checked daily for emergence of adults. At least 100 individuals (50 males and 50 females) from each isofemale line were then placed in containers (with an egg laying sheet and honey solution) for 3 days. Subsequently, the eggs were reared and the isofemale line continued as above.

In each generation the sex ratio of each line was recorded by counting the total number of male and females. The prevalence of *plutWB1 *was estimated by screening at least 10 males and 10 females per line. However, low numbers reduced sample size in two lines (17, 95) in generations one and two. The lines were kept in a controlled environment room, with a 16:8 light: dark cycle, at 23 C and 50–75% humidity.

### Statistical analysis of the phenotypic effects of *Wolbachia*

We first tested whether *plutWB1 *infection was associated with host sex ratio distortion and then whether there was evidence for male-killing. Since both sex ratio and infection status are binomial variables yielding proportion data, we arcsin transformed [arcsin(√*proportion (p)*] these variables. Data analysis was performed in Excel with the Analysis-It software.

We tested for departures from a 1:1 sex ratio in field populations using the Chi-squared test on data from individual and pooled valleys in the Cameron Highlands, Malaysia. The same test for deviation from 1:1 was also used for sex ratios of infected and uninfected isofemale lines.

We tested for a significant difference in sex ratio between infected and uninfected lab lines. We first used an unpaired t-test to examine if there was any significant difference in the sex ratio between infected and uninfected lines, using the total numbers of males and females in all nine generations for each line. We then examined whether infected lines varied across generations, by comparing the sex ratios of early (1 to 3), mid (4–6) and late (7 to 9) generations using a repeated measures ANOVA. Finally, we confirmed that there was no difference between the two infected lines using a paired t-test (*t *= 0.46, d.f. = 8, *P *> 0.05).

For infection frequency, we first confirmed that there was no difference between the two infected lines, using a paired t-test (*t *= 1.51, d.f. = 8, *P *> 0.05). This then allowed us to use the two lines as independent replicates in a repeated measures ANOVA to test if infection frequency changed between early, mid and late generations (see above). We conducted this analysis for males and females separately. Finally, we used a t-test on all males and all females to test for a sex difference in infection frequency. This focus on differences between the sexes is because a male-killing *Wolbachia *infection is expected to occur at (much) lower frequency in males than in females, unless effective host resistance has evolved.

## Abbreviations

(MP): Maximum parsimony; (NJ): neighbour-joining; (TBR): tree-bisection-reconnection.

## Authors' contributions

JMC conceived and AD designed the project. AD carried out the field and laboratory practical work and analysed the data. JMC and AD wrote the paper and both authors read and approved the final manuscript.
